# Health care costs, long-term survival, and quality of life following intensive care unit admission after cardiac arrest

**DOI:** 10.1186/cc6963

**Published:** 2008-07-18

**Authors:** Jürgen Graf, Cecile Mühlhoff, Gordon S Doig, Sebastian Reinartz, Kirsten Bode, Robert Dujardin, Karl-Christian Koch, Elke Roeb, Uwe Janssens

**Affiliations:** 1 Department of Anaesthesia and Intensive Care Medicine and Department of Cardiovascular Surgery, Philipps-University Marburg, Baldingerstrasse, 35043 Marburg, Germany; 2 Department of Dermatology, RWTH Aachen, University Hospital Aachen, Pauwelsstrasse 30, 52074 Aachen, Germany; 3 Department of Intensive Care, Royal North Shore Hospital and Senior Lecturer in Intensive Care, Northern Clinical School, Department of Medicine, University of Sydney, NSW 2006 Sydney, Australia; 4 Medical Clinic, St. Antonius Hospital Eschweiler, Dechant-Decker-Strasse, 52249 Eschweiler, Germany; 5 Zentrale Patientenaufnahme, Malteser-Krankenhaus St. Elisabeth, Kurfürstenstrasse 22, 52428 Jülich, Germany; 6 Medical Clinic I, Department of Cardiology and Intensive Care Medicine, RWTH Aachen, University Hospital Aachen, Pauwelsstrasse 30, 52074 Aachen, Germany; 7 Department of Medicine II, Head of Gastroenterology, Justus-Liebig-University Giessen, Paul-Meimberg-Strasse 5, 35385 Giessen, Germany

## Abstract

**Introduction:**

The purpose of this study was to investigate the costs and health status outcomes of intensive care unit (ICU) admission in patients who present after sudden cardiac arrest with in-hospital or out-of-hospital cardiopulmonary resuscitation.

**Methods:**

Five-year survival, health-related quality of life (Medical Outcome Survey Short Form-36 questionnaire, SF-36), ICU costs, hospital costs and post-hospital health care costs per survivor, costs per life year gained, and costs per quality-adjusted life year gained of patients admitted to a single ICU were assessed.

**Results:**

One hundred ten of 354 patients (31%) were alive 5 years after hospital discharge. The mean health status index of 5-year survivors was 0.77 (95% confidence interval 0.70 to 0.85). Women rated their health-related quality of life significantly better than men did (0.87 versus 0.74; *P *< 0.05). Costs per hospital discharge survivor were 49,952 €. Including the costs of post-hospital discharge health care incurred during their remaining life span, the total costs per life year gained were 10,107 €. Considering 5-year survivors only, the costs per life year gained were calculated as 9,816 € or 14,487 € per quality-adjusted life year gained. Including seven patients with severe neurological sequelae, costs per life year gained in 5-year survivors increased by 18% to 11,566 €.

**Conclusion:**

Patients who leave the hospital following cardiac arrest without severe neurological disabilities may expect a reasonable quality of life compared with age- and gender-matched controls. Quality-adjusted costs for this patient group appear to be within ranges considered reasonable for other groups of patients.

## Introduction

The annual incidence of sudden cardiac arrest in central Europe is approximately nine arrests per 10,000 inhabitants [1]. Thus, more than 600,000 people in Europe may be affected each year. Since the 1960s, immediate cardiopulmonary resuscitation (CPR) has been considered life-saving for sudden cardiac arrest [2], and following successful CPR, patients are routinely admitted to intensive care units (ICUs) to manage both the causes and acute sequelae. ICUs consume a large proportion of hospital budgets yet care for a minority of patients [3]. Longstanding economic constraints, present within all health care systems, create pressures to ration ICU care ethically [4]. Restricting the demands for futile medical services by limiting access to the ICU [5], at least for those patients likely to die anyway [6], has been proposed as a theoretical model to lower expenditures. In patients with sudden cardiac arrest, ICU and hospital lengths of stay are often protracted and incurred health care costs are high. Despite high short-term mortality and significant morbidity [7], long-term functional capacity for those surviving the initial hospitalization remains good [8]. Objective cost-outcome studies, integrating costs and quality-adjusted life years (QALYs) gained, are required to determine whether ICU admission constitutes a reasonable use of constrained resources in this patient population. To investigate the costs and long-term health status outcomes after CPR for out-of-hospital or in-hospital cardiac arrest, we conducted an individual patient-level assessment of health status at 5 years post-ICU discharge and combined these outcomes with a fully costed economic evaluation. All consecutive patients admitted to a single tertiary ICU were eligible for follow-up. We calculated the costs per survivor, costs per life year gained, and costs per QALY gained. A sensitivity analysis was conducted to model the impact of changes in utility (life years gained and health status index [HSI]) on the development of cost-outcome indices.

## Materials and methods

### Eligibility criteria

The study protocol was approved by the University Hospital of Aachen (Aachen, Germany) research ethics committee. Formal consent prior to contact for patient follow-up was not required since all patients who were contacted had the chance to refuse completion of the questionnaire. All patients admitted to the ICU of Medical Clinic I from 1 January 1999 to 31 December 2001, who received CPR for out-of-hospital or in-hospital cardiac arrest for any cause, were eligible for study entry. For the purpose of this study, CPR was defined as at least one cycle of chest compression and ventilation in patients with signs and/or symptoms of cardiac arrest. Patient care was at the discretion of the intensivist in charge without any explicit standard of care beyond the normal institutional standards and guidelines. Neither care of the patients nor end-of-life decision making was influenced by the study protocol at any time. Demographic data, admission diagnoses, lengths of ICU and hospital stays, and ICU and hospital mortality rates were collected prospectively. Severity of illness was classified using the Simplified Acute Physiology Score II (SAPS II) [9] for the initial 24 hours after admission to the ICU. The simplified Therapeutic Intervention Scoring System (TISS-28) [10] and the Sequential Organ Failure Assessment (SOFA) [11] were collected daily, and the total maximum SOFA (TMS) was calculated at the end of the ICU stay [12].

### Outcome assessment

Health-related quality of life (HRQL) was obtained 5 years after ICU discharge using a regular mail formal letter, including a return envelope, containing the validated German interview form [13] of the Medical Outcome Survey Short Form-36 questionnaire (SF-36) self-report form [14]. In addition, the questionnaire assessed employment and marital status, dependency, re-hospitalizations, patients' recollection of their ICU stay, and their willingness to undergo critical care, if necessary, again. If patients did not respond to the questionnaire and telephone contact could not be established, the family doctor and/or relatives of the patients were contacted to provide the correct address of the patient or to confirm death after hospital discharge. Patients who could not be contacted, but were known to be alive, were considered lost to follow-up with regard to HRQL. Normative HRQL data, including apparently healthy controls [15] and patients with acute and chronic diseases [16], are available for different age groups of the German population. An HSI, which represents overall quality of life relative to an age-matched reference group, was calculated for each patient using the SF-36 results. The HSI is a weight ranging from 0 (indifference between life and death) to 1 (perfect health) and was calculated for each patient as the mean of the individual domain indices for the eight domains of the SF-36 (that is, by dividing the individual patient result for a particular domain by the domain mean of the normative data obtained from apparently healthy Germans [15], summing each domain index, and dividing by the number of domains). The HSI multiplied by life years gained results in QALYs [17]. Patients discharged from the hospital with a Glasgow Coma Scale (GCS) score of below 6 points (that is, severely neurologically disabled patients) were analyzed separately since formal objective quality-of-life assessment was not possible.

### Costing methodology

The costing methodology in this study is a modification of the 'bottom-up' approach [18,19]. Costs were not limited to index hospital and ICU admission costs. From the perspective of the society's health care system, we included post-hospital discharge health care costs and used a 3% annual discount rate [20]. Other costs to society, such as time lost from work, were not considered. To consider differences in the complexity of the individual patient, ICU costs were divided into patient-specific (variable) and non-patient-specific (fixed) costs. Non-patient-specific costs were calculated on a patient-day basis, whereas patient-specific costs were directly attributed to the individual patient. Labor costs were divided into patient-specific and non-patient-specific costs. Wages of nurses and physicians on duty were allocated to patient-specific costs and distributed according to the patient's TISS-28 score to account for patient differences in therapeutic activities. 'Backup staff' (that is, vacancies and off-shift) costs were calculated as day-related non-patient-specific costs. The costs for radiology, clinical chemistry, pathology, and microbiology were calculated according to the German regulation of charges for physicians [21]. Non-patient-related administrative costs were calculated as a share of hospital costs in relation to the size of the unit (energy, heating, and maintenance) and the number of patients and staff (administrative costs).

### Cost-outcome descriptions

This study presents both the outcomes and costs associated with this patient cohort. Cost-outcome descriptions are presented as costs per survivor, costs per long-term (5 years) survivor, costs per life year gained, and costs per QALY gained. To obtain costs per survivor and costs per long-term survivor, total ICU, hospital, and post-hospital discharge health care costs were divided by the number of patients who survived hospital discharge and the number of patients remaining alive at 5 years post-hospital discharge. Age- and gender-specific expected annual post-hospital discharge health care costs were obtained from the German Ministry of Health (that is, for males: 30 to 44 years, 1,270 € per year; 45 to 64 years, 2,760 € per year; 65 to 85 years, 5,830 € per year; for females: 30 to 44 years, 1,840 € per year; 45 to 64 years, 3,160 € per year; 65 to 85 years, 6,250 € per year as of the year 2004 [22]). Post-hospital nursing home costs were estimated for all patients with a GCS score below 6 points as a monthly average of 2,700 € (as of the year 2004 [22]). A yearly discount of 3% was subtracted or added for the years before or following 2004, respectively. To estimate the cost per life year gained, the total costs were divided by the total estimated life years gained. Life years gained was calculated as the total life years of follow-up time observed in all patients post-discharge plus the estimated remaining life span of the patients alive at 5 years. The estimated remaining life span was calculated conservatively based on an average age-adjusted life expectancy of 80.5 years for the male population and 84.3 years for the female population (for a 65-year-old person as of the year 2002, German Ministry of Health [22]). Cost-utility descriptions were generated via an HSI adjustment of life years gained. The number of QALYs gained is the product of the number of life years gained multiplied by HSI. Note that the HSI could be obtained only for patients surviving at 5-year follow-up; thus, only their life years gained were adjusted. This is a conservative approach since patients who were discharged alive, but did not survive to year 5, may have gained quality survival time.

### Statistics

All variables were tested for the assumption of normality using the Kolmogorov-Smirnov test. Descriptive statistics are reported as mean and 95% confidence interval (CI), except when stated otherwise. The Student *t *test was used for comparisons of means of normally distributed data. A non-parametric rank test (Mann-Whitney *U *test) was applied in case of non-normally distributed data. Categorical data were tested using the χ^2 ^statistics with Yates correction when appropriate. Internal consistency of the various domains of the SF-36 was assessed using the Cronbach's alpha coefficient. A Cronbach's alpha exceeding 0.7 is considered to demonstrate acceptable agreement [23]. In a two-way sensitivity analysis, both HSI and incremental life years saved for all 5-year survivors who completed the questionnaire were increased and decreased by 25% and 50%, respectively. Costs were adjusted considering the changes in post-hospital health care expenses owing to a longer or shorter remaining life span. All statistical tests were two-sided, and a significance level (*P *value) of less than 0.05 was applied, except when stated otherwise. Data were analyzed using SPSS 12.0 (SPSS Inc., Chicago, IL, USA).

## Results

### Patient outcomes

**Figure 1:**
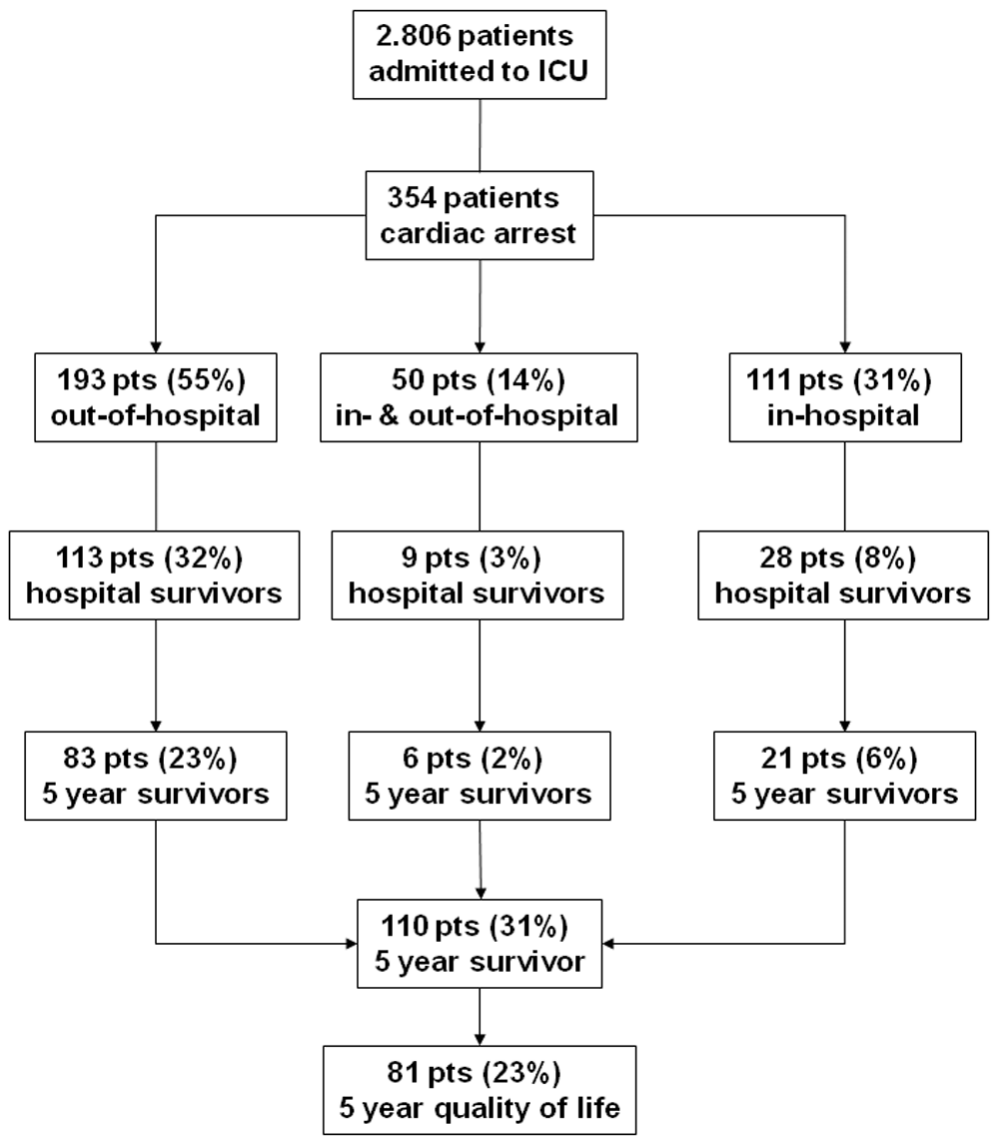
From January 1999 to December 2001, a total of 2,806 patients were admitted to the medical intensive care unit (ICU). Of those patients, 354 (13%) had a cardiac arrest with subsequent cardiopulmonary resuscitation out of hospital, in hospital, or both and thus qualified for study entry.

**Figure 2:**
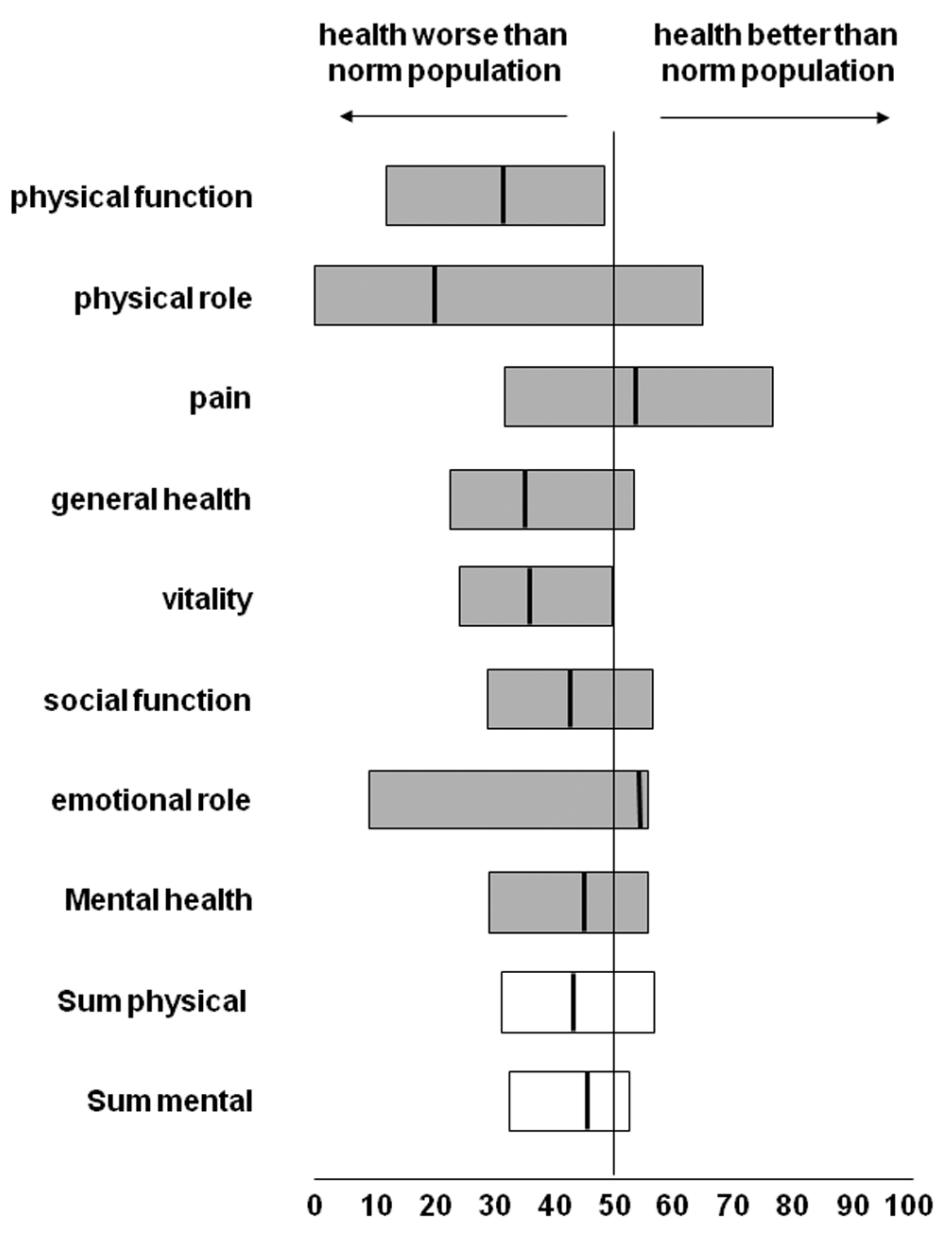
Medical Outcome Survey Short Form-36 questionnaire (SF-36) of 81 long-term survivors after cardiopulmonary resuscitation. Each scale is normalized to a mean of 50, which is considered normal on the basis of comparison of SF-36 scores in a general gender- and age-matched German control (norm population). The vertical line represents the median, and the left and right limits of the boxes represent the quartiles. Cronbach's alpha coefficient exceeded 0.7 in all domains, demonstrating acceptable agreement of the various items.

Of 354 patients admitted to the ICU with cardiac arrest, 204 patients (58%) died prior to discharge from the hospital, either during their ICU stay (n = 171) or later on the ward (n = 26), not including 7 patients who were discharged with severe disabilities (that is, a GCS score below 6 points). Of the 150 patients (42%) remaining, 40 patients died before year 5, leaving 110 patients (31%) eligible to be surveyed at 5 years. Twenty patients declined to respond to the HRQL survey at 5-year follow-up, and 9 patients were known to be alive but were otherwise lost to follow-up. Eighty-one patients (74% of all 5-year survivors) completed the entire questionnaire. Complete demographic information is presented in Table 1. The 29 patients who were unavailable for final follow-up stayed significantly longer in the ICU and hospital compared with the cohort completing the questionnaire. Demographic data, severity of illness on admission (SAPS II), or morbidity (TMS and TISS-28) did not differ between the two groups. The final cost-utility description is based on the 81 complete data sets only (Figure 1). Prior to cardiac arrest, 60 patients (74%) lived self-supported, a status that was maintained by 56 patients (68%) 5 years later. The number of patients living in their own home with some level of support increased from 8 patients (10%) to 13 patients (16%). After hospital discharge, only a minority of patients relied on daily custodial support (3 patients, 4%) or lived in nursing homes (6 patients, 5%). Five years after hospital discharge, 13 (16%) survivors were employed, 13 (16%) were early retired, and 48 (59%) were regularly retired due to age. Forty-eight (59%) survivors were re-hospitalized during the 5-year follow-up (23 survivors once, 5 twice, and 5 three times). Twenty-two (27%) survivors recalled unpleasant or alarming memories with regard to their ICU stay. Sixty-nine (85%) reported that they would undergo intensive care again if necessary. HRQL is displayed in Figure 2. Except pain, emotional role function, and mental health, all other items were rated somewhat lower than in an age- and gender-matched population of apparently healthy Germans [15]. The 81 long-term survivors reached a mean HSI of 0.77 (95% CI 0.70 to 0.85). Women rated their HRQL significantly better than men did (HSI 0.87 versus 0.74; *P *< 0.05). There were no differences in age, severity of illness, ICU and hospital lengths of stay, or admission diagnosis between men and women. The individual items of pain, emotional role function, and physical role function were rated superior by women (*P *< 0.05) after 5 years.

**Table 1:** Demographic data, admission diagnosis, severity of illness, and morbidity of all patients admitted to the intensive care unit from 1999 to 2001 with cardiac arrest (n = 354)

	Cardiac arrest (n = 354)	Hospital non-survivors (n = 204)	Completed questionnaire (n = 81)	GCS score below 6 points (n = 7)
Age in years, mean ± SD	66 ± 13	68 ± 12	61 ± 13	61 ± 12
Median (25th/75th percentile)	68 (60/75)	70 (62/77)	61 (53/72)	64 (50/68)
Gender male/female, number (percentage)	252/102 (71/29)	148/56 (73/27)	57/24 (70/30)	6/1 (86/14)
ICU stay in days, mean ± SD (minimum-maximum)	9 ± 16 (1–113)	8 ± 14 (1–98)	7 ± 11 (1–78)	17 ± 23 (2–67)
Median (25th/75th percentile)	3 (1/9)	4 (1/9)	3 (2/7)	6 (2/18)
Hospital stay in days, mean ± SD (minimum-maximum)	25 ± 28 (1–176)	4 ± 18 (1–150)^a^	19 ± 18 (1–103)	35 ± 30 (2–101)
Median (25th/75th percentile)	15 (5/33)	NA	14 (10/22)	26 (15/38)
SAPS II, mean ± SD	47 ± 23	58 ± 19^a^	38 ± 20	47 ± 21
Median (25th/75th percentile)	45 (31/63)	58 (44/71)^a^	33 (23/53)	53 (24/66)
SAPS II PRM as a percentage, mean ± SD	42 ± 33	58 ± 30^a^	42 ± 33	45 ± 36
Median (25th/75th percentile)	35 (12/74)	64 (33/85)^a^	14 (5/52)	53 (6/78)
Simplified TISS-28 day 1, mean ± SD	34 ± 11	36 ± 10	31 ± 8	37 ± 3
Median (25th/75th percentile)	34 (28/40)	36 (28/43)	32 (26/37)	37 (34/40)
TMS, mean ± SD	9.6 ± 5.2	11.8 ± 4.5^a^	6.3 ± 5.0	7.7 ± 5.3
Median (25th/75th percentile)	11 (5/13)	13 (10/15)^a^	5 (2/11)	9 (1/10)

Hospital non-survivors were more severely ill (SAPS II; *P *< 0.05) and exhibited significantly more organ dysfunctions (TMS; *P *< 0.05) compared with the patients completing the follow-up of 5 years. Age is given as of the day of ICU admission. ^a^Significant difference between all patients and those who survived the hospital stay (*P *< 0.05). GCS, Glasgow Coma Scale; ICU, intensive care unit; NA, not applicable; PRM, predicted risk of mortality; SAPS II, Simplified Acute Physiology Score II; SD, standard deviation; TISS-28, Therapeutic Intervention Scoring System; TMS, total maximum Sequential Organ Failure Assessment.

### Costs

The total ICU costs for all 354 patients with cardiac arrest amounted to 6,312,700 € (Table 2). The costs for the ward stay after ICU discharge accounted for 295 € per patient per day. The total hospital length of stay was 7,544 days. The total hospital costs, including the ICU stay, amounted to 7,492,771 €. The ICU stay accounted for 84% of total in-hospital costs.

**Table 2:** Intensive care unit (ICU) costs incurred for all 354 patients separated into total ICU costs per patient and daily ICU costs per patient

	Mean	Range	95% confidence interval
Total ICU costs per patient	17,832 €	1,708 to 181,500 €	15,280 to 20,390 €
Daily ICU costs per patient	2,693 €	656 to 5,856 €	2,555 to 2,832 €

Post-hospital costs of future health care utilization for all 150 patients discharged alive were estimated to be 16,856,851 €, based on the projected remaining life span of a total 2,409 person-years. For the 110 patients known to be alive at 5 years, costs of future health care utilization after hospital discharge would amount to 15,615,920 €, based on the projected remaining life span of 2,354 years. The estimated long-term survival of the seven patients with a GCS score below 6 points, including the incurred ICU, hospital, and post-hospital costs, is displayed in Table 3. The post-hospital costs of future health care utilization were estimated to be 1,179,329 €, based on the projected remaining life span of a total 141 person-years. Including average nursing home costs of 2,700 € per month, total post-hospital costs would amount to 5,747,729 €. For the 110 patients known to be alive at 5 years, costs of future health care utilization after hospital discharge would amount to 15,615,920 €, based on the projected remaining life span of 2,354 years.

**Table 3:** Calculated ICU and hospital costs and estimated post-hospital costs incurred for the seven patients with a Glasgow Coma Scale score below 6 points

	Mean	SD	Median (interquartile range)
Daily ICU costs per patient	2,285 €	638 €	2,012 € (1,887 €/2,934 €)
Total hospital costs per patient	35,910 €	37,579 €	18,297 € (14,648 €/33,253 €)
Post-hospital costs per patient	132,565 €	59,878 €	122,945 € (91,094 €/186,888 €)
Nursing home costs per patient	654,480 €	362,880 €	534,600 € (405,000 €/988,200 €)

Costs are based on a projected mean of 20 life years gained per patient (median 16.5 years [12.5/30.5]) and a cumulative survival of 141.3 years. ICU, intensive care unit; SD, standard deviation.

### Costs per survivor and costs per long-term survivor

The costs per hospital survivor were calculated to be 49,952 € (that is, 7,492,771 € total hospital costs divided by 150 hospital discharge survivors). For the 110 patients surviving 5 years, initial ICU and hospital costs per long-term survivor were 68,116 € (that is, 7,492,771 € total hospital costs divided by 110 5-year survivors).

### Costs per life years gained

The 150 hospital discharge survivors were calculated, using life table methods, to have an estimated total remaining life span of 16 years per patient (95% CI 14 to 18 years) at the time of hospital discharge, which provides an additional 2,409 person-years. The estimated age-adjusted post-hospital discharge health care costs for these patients were calculated to be 6,997 € per person-year. Considering all hospital discharge survivors, the costs per life year gained were 10,107 € (that is, 7,492,771 € total hospital costs plus 16,856,851 € post-hospital discharge health care costs divided by 2,409 person-years gained). Including the costs incurred for the seven severely disabled patients (that is, applying life table methods to 157 hospital survivors), these costs increased to 11.757 € per life year gained. Considering only long-term survivors, patients alive at 5 years were calculated to have an estimated total remaining life span of 15 years per patient (95% CI 13 to 18 years) which, including the 5 years already survived per patient, accounts for a total additional 2,354 person-years. The age-adjusted post-hospital health care costs for these patients were calculated to be 6,634 € per remaining life year for a total of 15,615,920 €. Ignoring life years gained from patients who died before 5 years post-discharge, the costs per life year gained for long-term survivors were calculated as 9,817 € (that is, 7,492,771 € total hospital costs plus 15,615,920 € post-hospital discharge health care costs divided by 2,354 person-years gained). Again, including the seven patients with a GCS score below 6 points, costs per life year gained account for 11,566 €. It is important to present these long-term survivors separately since they represent the total sampling frame from which HRQL information could be obtained at 5 years.

### Costs per quality-adjusted life year

**Figure 3:**
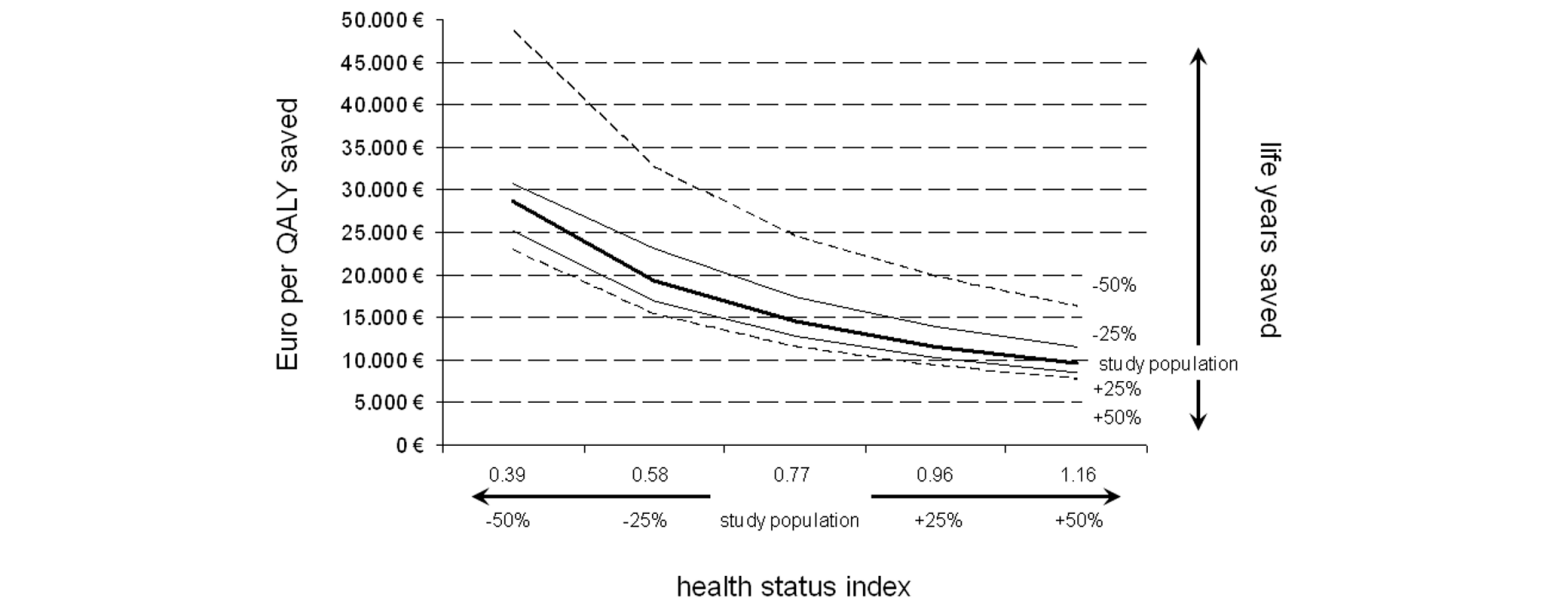
Two-way sensitivity analysis depicting costs per quality-adjusted life year (QALY) saved considering both an increase and a decrease in health status index 5 years post-intensive care unit of 25% and 50%, respectively (bold solid line). Moreover, the remaining life years were modelled, again considering an increase and a decrease of 25% (fine line) and 50% (dashed line), respectively.

Information on HRQL, and thus HSI 5 years after hospital discharge, was available in 81 patients (74% of all 5-year survivors). These 81 patients had an estimated average remaining life span of 21 years per patient (95% CI 18 to 24 years) for a total of 1,709 person-years and a calculated average HSI of 0.77 (95% CI 0.70 to 0.85). In this group, total post-hospital health care costs were 11,572,491 €. Incremental costs per life year gained thus amounted to 11,156 €. The estimated remaining life span of 1,709 years multiplied by the HSI of 0.77 translates into 1,316 QALYs, averaging 14,487 € per QALY gained. A simulation, including the 20 patients who declined responses to the HRQL survey and the 9 patients who were lost during follow-up, revealed an estimated total remaining life span of up to 2,327 life years (average 21 years per patient, 95% CI 19 to 24 years). Assuming an HSI of 0.75, which is comparable to those patients who completed the HRQL questionnaire, an additional 1,766 QALYs would have been gained. Thus, incremental costs per life year gained for all 110 5-year survivors would have amounted to 9,931 €, with 13,085 € per QALY gained. Figure 3 illustrates the influence of changes in utility (life years gained and HSI) and costs for the 81 patients with completed 5-year HRQL follow-up.

## Discussion

Patients who survive cardiac arrest are often considered to have a grim prognosis and discussion ensues as to whether they should be universally welcomed to the ICU: costs are expected to be high and benefits are expected to be moderate at best. Herein, we present information from a cohort of patients with HRQL obtained at 5 years following cardiac arrest and subsequent CPR. We found that HRQL 5 years after hospital discharge was only slightly lower than age- and gender-matched apparently healthy German controls. In addition, both the reported survival (ICU and hospital stays) [24] and HRQL of our cohort did not differ significantly when compared with publications based on similar patient populations (in-hospital and out-of-hospital cardiac arrest) [8], other medical ICU patients [25,26], or ICU patients with sepsis [27]. We followed our patients for 5 years to allow sufficient recovery time before the assessment of health status. This time frame was selected because the slope of the survival curve can be expected to proceed in parallel with that of a control population and it is unlikely that patients' HRQL attributable to the index hospitalization will improve [25,26,28,29]. These assumptions permit the prediction of the cohort's remaining life span based on data from the German Census Bureau and the calculation of a valid HSI by close approximation. Mean patient costs per ICU day were twice those obtained for average ICU patients admitted to the same institution (2,693 € compared with 1,334 € in less severely ill patients [30]). Consequently, costs per 5-year survivor were also considerably higher than the average ICU patient (68,116 € versus 14,130 € [26]). This is attributable to both higher costs per ICU stay and higher short-term (that is, ICU and hospital) mortality in the cohort of patients with cardiac arrest; however, the health status outcomes and costs per life year saved and per QALY of our patients compared favorably with general cardiovascular and pulmonary ICU patient populations from the same ICU [26]. They also compare favorably with cost-outcome profiles of a variety of other interventions routinely undertaken in the critically ill as well as non-ICU patients (Table 4). Our findings are robust under a wide range of sensitivity analyses adjusting for patient mortality rates and HSI projected over the estimated remaining life expectancy. In simulation, significant cost increases per life year gained or per QALY gained were observed only after the mortality was increased to over 50% (Figure 3) or by decreasing the HSI below 0.58.

**Table 4:** Arbitrary selection of trials investigating cost per quality-adjusted life year for a variety of interventions in the critically ill and in non-ICU patients

Critically ill	Costs per quality-adjusted life year
Hamel, *et al*. (2000) [36]	Low-risk group (likelihood of surviving 2 months, >70%): 28,889 €
Mechanical ventilation for acute respiratory failure due to pneumonia or Adult Respiratory Distress Syndrome versus mechanical ventilation withheld	Medium-risk group (likelihood of surviving 2 months, 51% to 70%): 43,832 €
	High-risk group (likelihood of surviving 2 months, <50%): 109,582 €
Hamel, *et al*. (1997) [37]	Average costs: 143,742 €
Initiating dialysis and continuing aggressive care in seriously ill patients versus withholding renal support therapy	Best prognostic category: 69,404 €
	Worst prognostic category: 307,329 €
Paniagua, *et al*. (2002) [38]	Quality of life estimated: 84,365 €
Cardiopulmonary resuscitation for in-hospital cardiac arrest in octogenarians followed by aggressive treatment	
Non-ICU patients	
CDC Diabetes Cost-effectiveness Group (2002) [39]	Intensive glycemic control: 42,463 €
Reducing complications in patients with type 2 diabetes using various interventions	Reducing serum cholesterol level: 53,242 €
	Intensified hypertension control saves 2,010 €
Wonderling, *et al*. (2004) [40]	13,311 €
Acupuncture for chronic headache in primary care versus usual care only	
Brunner-La Rocca, *et al*. (2007) [41]	40,467 €
Drug-eluting stents versus bare-metal stents in percutaneous coronary interventions	

All costs are converted in 2004 Euro (€) with 3% annual discount (1 US $ = 0.81 Euro; 1 British pound = 1.45 Euro). Note that underlying methods for assessing costs and relevant outcomes as well as follow-up period vary considerably. ICU, intensive care unit.

There are several unique aspects of our study which should be considered further. First, health care expenditures do not usually end with hospital discharge, especially for critically ill patients. In our cohort, the estimated costs incurred for post-hospital discharge health care services of all 5-year survivors surpassed their initial ICU and hospital costs by more than twofold. It is important to point out that, in the absence of specific data for patients following cardiac arrest, we based these estimates on average age- and gender-adjusted health care utilization costs provided by the German Bureau of Census. Since the majority of the patients in our cohort were readmitted to hospital at least once during the 5-year follow-up, true long-term health care costs of this patient group may be above the expected averages reported by the Bureau of Census; however, there is no reason to expect that they are above the average that could be expected for other ICU survivors. It is important to note that we did not consider the potential overall costs for the society as a whole (earlier retirement, higher employees' sickness rates, and so on). The compilation of such data is largely based on estimations and was beyond the scope of this study. Second, the expected remaining life span of our 5-year survivors was also based on census data. Since the best estimates suggest that the hazard of death for survivors of in-hospital arrest parallels the appropriately age- and gender-matched general population after 2 years [31], it is reasonable to assume that the hazard of death for our patient cohort parallels the appropriate age- and gender-matched population. Thus, the application of standard life table (actuarial) methods, using population-based life expectancy tables, is likely a reasonable approach for estimating remaining life expectancy. Third, there is a dilemma with patients surviving hospital discharge with severe neurological deficits (as defined here with a GCS score of less than 6 points). What is the quality of life or HSI of such a patient? To the best of our knowledge, we cannot judge. On a utility scale from zero (representing death) to one (representing perfect health), such patients definitely do not represent one but are most likely not zero either. We therefore calculated costs of these patients separately for life years gained but did not include any utility measures. In these patients, we did not discount life expectancy although one may expect shorter overall survival in this group [32]. Thus, costs incurred for this subgroup as well as for the whole population are most likely overestimated. Fourth, both socioeconomic status and occupational class may affect patients' perception of quality of life, with patients belonging to lower status groups reporting a quicker decline in self-reported health [33]. However, we did not assess socioeconomic status or occupational class of our patients and thus could not adjust our data. Finally, we should point out that the Utstein style protocol [34] of basic and advanced life support was not available in our patient population. Although this would have been desirable for auditing resuscitation efforts, it was deemed of secondary interest since our study focused on outcomes and costs, not quality of care.

## Conclusion

Despite some restrictions that emerge owing to the methodologic complexities inherent in any cost-outcome description conducted in ICU patients [20,35], we found that the costs per life year and per QALY gained for patients with cardiac arrest who require ICU admission are reasonable (approximately 9,930 € and 13,000 €, respectively). Moreover, our data highlight the somewhat skewed notion that extreme expenses result from the care of patients who have undergone basic life support following cardiac arrest. Although it may be true that patients with cardiac arrest incur considerable costs and resource consumption, the trade-off between input and output, costs and outcome, justifies such resource allocation, at least in comparison with other ICU patient groups. We believe our study is the first to demonstrate that patients who leave the hospital following cardiac arrest without severe neurological disabilities may expect fair long-term survival and quality of life for reasonable expenses to the health care system.

## Key messages

• Patients who leave the hospital following cardiac arrest without severe neurological disabilities may expect fair long-term survival and a good quality of life.

• Costs per life year gained and costs per quality-adjusted life year in survivors after cardiac arrest are acceptable.

• Expenses to the health care system are reasonable compared with other interventions carried out in both intensive care unit (ICU) and non-ICU patients.

## Abbreviations

CI = confidence interval; CPR = cardiopulmonary resuscitation; GCS = Glasgow Coma Scale; HRQL = health-related quality of life; HSI = health status index; ICU = intensive care unit; QALY = quality-adjusted life year; SAPS II = Simplified Acute Physiology Score II; SF-36 = Medical Outcome Survey Short Form-36 questionnaire; SOFA = Sequential Organ Failure Assessment; TISS-28 = Therapeutic Intervention Scoring System; TMS = total maximum Sequential Organ Failure Assessment.

## Competing interests

The authors declare that they have no competing interests.

## Authors' contributions

JG was responsible for study conception, the development of the design of the study, and analyzed the raw data. CM helped to develop the design of the study. K-CK helped to develop the design of the study and to analyze the raw data. GSD, ER, and UJ helped to analyze the raw data. All authors contributed to the interpretation of data, the drafting of the article, and critical appraisal of all draft versions for important intellectual content. No other contributor or professional medical writer was involved. All authors give final approval of the version to be published.

## References

[B1] Gorgels AP, Gijsbers C, de Vreede-Swagemakers J, Lousberg A, Wellens HJ (2003). Out-of-hospital cardiac arrest – the relevance of heart failure. The Maastricht Circulatory Arrest Registry. Eur Heart J.

[B2] Kouwenhoven WB, Jude JR, Knickerbocker GG (1960). Closed-chest cardiac massage. JAMA.

[B3] Kuckelt W, Reis Miranda D, Ryan DW, Schaufeli WB, Fidler V (1998). Notes in intensive care medicine in Europe. Intensive care medicine in Germany. Organisation and Management of Intensive Care A Prospective Study in 12 European Countries.

[B4] Bion J (1995). Rationing intensive care. BMJ.

[B5] Fries JF, Koop CE, Beadle CE, Cooper PP, England MJ, Greaves RF, Sokolov JJ, Wright D (1993). Reducing health care costs by reducing the need and demand for medical services. N Engl J Med.

[B6] Bayer R, Callahan D, Fletcher J, Hodgson T, Jennings B, Monsees D, Sieverts S, Veatch R (1983). The care of the terminally ill: morality and economics. N Engl J Med.

[B7] The Hypothermia after Cardiac Arrest Study Group (2002). Mild therapeutic hypothermia to improve the neurologic outcome after cardiac arrest. N Engl J Med.

[B8] Bunch TJ, White RD, Gersh BJ, Meverden RA, Hodge DO, Ballman KV, Hammill SC, Shen WK, Packer DL (2003). Long-term outcomes of out-of-hospital cardiac arrest after successful early defibrillation. N Engl J Med.

[B9] Le Gall JR, Lemeshow S, Saulnier F (1993). A new Simplified Acute Physiology Score (SAPS II) based on a European/North American multicenter study. JAMA.

[B10] Moreno R, Morais P (1997). Validation of the simplified therapeutic intervention scoring system on an independent database. Intensive Care Med.

[B11] Vincent JL, Moreno R, Takala J, Willatts S, De Mendonca A, Bruining H, Reinhart CK, Suter PM, Thijs LG (1996). The SOFA (Sepsis-Related Organ Failure Assessment) score to describe organ dysfunction/failure. On behalf of the Working Group on Sepsis-Related Problems of the European Society of Intensive Care Medicine. Intensive Care Med.

[B12] Janssens U, Graf C, Graf J, Radke PW, Königs B, Koch KC, Lepper W, vom Dahl J, Hanrath P (2000). Evaluation of the SOFA score: a single centre experience of a medical intensive care unit in 303 consecutive patients with predominantly cardiovascular disorders. Intensive Care Med.

[B13] Bullinger M (1995). German translation and psychometric testing of the SF-36 health survey: preliminary results from the IQOLA project. Soc Sci Med.

[B14] Ware JE, Sherbourne CD (1992). The MOS 36-item short-form health survey (SF-36): I. Conceptual framework and item selection. Med Care.

[B15] Ellert U, Bellach B-M (1999). Der SF-36 im Bundes-Gesundheitssurvey – Beschreibung einer Stichprobe [German Health Survey and the SF-36: Description of a Control Sample.]. Gesundheitswesen.

[B16] Bullinger M, Kirchberger I (1998). SF-36 Fragebogen zum Gesundheitszustand [SF-36 Health-related Quality of Life Questionnaire].

[B17] Torrance GW (1997). Preferences for health outcomes and cost-utility analysis. Am J Manag Care.

[B18] Edbrooke DL, Hibbert CL, Ridley S, Long T, Dickie H (1999). The development of a method for comparative costing of individual intensive care units. The intensive care working group on costing. Anaesthesia.

[B19] Jegers M, Edbrooke DL, Hibbert CL, Chalfin DB, Burchardi H (2002). Definitions and methods of cost assessment: an intensivist's guide. ESICM section on health research and outcome working group on cost effectiveness. Intensive Care Med.

[B20] Members of the Second American Thoracic Society Workshop on Outcomes Research (2002). Understanding costs and cost-effectiveness in critical care. Am J Respir Crit Care Med.

[B21] Krimmel L, Hess R, Kleinken B, Warlo HJ (1996). Kommentar zur Gebührenordnung für Ärzte [Comments of the Physician Fee Schedule]. Kommentar zur Gebührenordnung für Ärzte.

[B22] Gesundheitsberichterstattung des Bundes. http://www.gbe-bund.de.

[B23] Bland JM, Altman DG (1997). Statistics notes: Cronbach's alpha. BMJ.

[B24] Nolan JP, Laver SR, Welch CA, Harrison DA, Gupta V, Rowan K (2007). Outcome following admission to UK intensive care units after cardiac arrest: a secondary analysis of the ICNARC case mix programme database. Anaesthesia.

[B25] Graf J, Koch M, Dujardin R, Kersten A, Janssens U (2003). Health-related quality of life before, one month and nine months after intensive care in medical cardiovascular patients. Crit Care Med.

[B26] Graf J, Wagner J, Graf C, Koch KC, Janssens U (2005). Five-year survival, quality of life, and individual costs of 303 consecutive medical intensive care patients – a cost-utility analysis. Crit Care Med.

[B27] Heyland DK, Hopman W, Coo H, Tranmer J, McColl MA (2000). Long-term health-related quality of life in survivors of sepsis. Short form 36: a valid and reliable measure of health-related quality of life. Crit Care Med.

[B28] Niskanen M, Kari A, Halonen P, the Finnish ICU Study Group (1996). Five-year survival after intensive care – comparison of 12180 patients with the general population. Crit Care Med.

[B29] Harve H, Tiainen M, Poutiainen E, Maunu M, Kajaste S, Roine RO, Silfvast T (2007). The functional status and perceived quality of life in long-term survivors of out-of-hospital cardiac arrest. Acta Anaesthesiol Scand.

[B30] Graf J, Graf C, Janssens U (2002). Analysis of resource use and cost-generating factors in a German medical intensive care unit employing the Therapeutic Intervention Scoring System (TISS-28). Intensive Care Med.

[B31] Kalbaga A, Kotyraa Z, Richards M, Spearpoint K, Brett SJ (2006). Long-term survival and residual hazard after in-hospital cardiac arrest. Resuscitation.

[B32] The Multi-Society Task Force on PVS (1994). Medical aspects of the persistent vegetative state – second of two parts. N Engl J Med.

[B33] Chandola T, Ferrie J, Sacker A, Marmot M (2007). Social inequalities in self reported health in early old age: follow-up of prospective cohort study. BMJ.

[B34] Recommended Guidelines for Uniform Reporting of Data From Out-of-Hospital Cardiac Arrest (New Abridged Version) (1992). The "Utstein Style". The European Resuscitation Council, American Heart Association, Heart and Stroke Foundation of Canada, and Australian Resuscitation Council. Br Heart J.

[B35] Siegel JE, Weinstein MC, Russell LB, Gold MR (1996). Recommendations for reporting cost-effectiveness analyses. Panel on cost-effectiveness in health and medicine. JAMA.

[B36] Hamel MB, Phillips RS, Davis RB, Teno J, Connors AF, Desbiens N, Lynn J, Dawson NV, Fulkerson W, Tsevat J (2000). Outcomes and cost-effectiveness of ventilator support and aggressive care for patients with acute respiratory failure due to pneumonia or acute respiratory distress syndrome. Am J Med.

[B37] Hamel MB, Phillips RS, Davis RB, Desbiens N, Connors AF, Teno JM, Wenger N, Lynn J, Wu AW, Fulkerson W, Tsevat J (1997). Outcomes and cost-effectiveness of initiating dialysis and continuing aggressive care in seriously ill hospitalized adults. SUPPORT investigators. Study to understand prognoses and preferences for outcomes and risks of treatments. Ann Intern Med.

[B38] Paniagua D, Lopez-Jimenez F, Londono JC, Mangione CM, Fleischmann K, Lamas GA (2002). Outcome and cost-effectiveness of cardiopulmonary resuscitation after in-hospital cardiac arrest in octogenarians. Cardiology.

[B39] CDC Diabetes Cost-effectiveness Group (2002). Cost-effectiveness of intensive glycemic control, intensified hypertension control, and serum cholesterol level reduction for type 2 diabetes. JAMA.

[B40] Wonderling D, Vickers AJ, Grieve R, McCarney R (2004). Cost effectiveness analysis of a randomised trial of acupuncture for chronic headache in primary care. BMJ.

[B41] Brunner-La Rocca HP, Kaiser C, Bernheim A, Zellweger MJ, Jeger R, Buser PT, Osswald S, Pfisterer M (2007). Cost-effectiveness of drug-eluting stents in patients at high or low risk of major cardiac events in the Basel Stent KostenEffektivitäts Trial (BASKET): an 18-month analysis. Lancet.

